# Ataque Isquêmico Transitório em Paciente com *Kinking* Vascular de Subclávia e de Carótida Comum Direitas - Um Relato de Caso

**DOI:** 10.36660/abc.20230520

**Published:** 2024-07-31

**Authors:** Katia Gleicielly Frigotto, Washington Luiz Batista da Costa, Lais Antonucci Ferreira, Daniela Roberta Alves Silva, Giovana Salviano Braga Garcia, Luciano de Figueiredo Aguiar, Bruno de Souza Paolino

**Affiliations:** 1 Hospital São Lucas Copacabana Rio de Janeiro RJ Brasil Hospital São Lucas Copacabana, Rio de Janeiro, RJ – Brasil; 2 Universidade do Grande Rio Professor José de Souza Herdy Rio de Janeiro RJ Brasil Universidade do Grande Rio Professor José de Souza Herdy, Rio de Janeiro, RJ – Brasil

**Keywords:** Doenças vasculares, Transtornos Cerebrovasculares, Ataque Isquêmico Transitório

## Introdução

O *kinking* vascular é uma alteração angular vascular em que se forma um ângulo agudo em uma artéria.^1^ Causas embriogênicas e adquiridas podem levar a essa alteração. Estudos indicam que a aterosclerose, a hipertensão e o envelhecimento desempenham um papel significativo na produção de anormalidades carotídeas.^2^ Outras causas, como trauma, etiologia congênita ou doenças inflamatórias, são raras e geralmente associadas à aterosclerose.^3^ Os fatores congênitos tendem a se tornar clinicamente significativos na idade avançada, agravados por hipertensão arterial sistêmica (HAS), diabetes mellitus (DM), dislipidemia, tabagismo, e doenças reumatológicas e cardíacas.^2^

Existem dois tipos de *kinking*. O primeiro é congênito, mais comum em mulheres no final dos 40 anos e início dos 50 anos, e clinicamente significativo na idade avançada. O segundo tipo está ligado a fatores de risco ateroscleróticos, como HAS, dislipidemia, DM e tabagismo.^1^ No entanto, o papel das anormalidades carotídeas na produção de sintomas isquêmicos é difícil de determinar devido à frequente associação com lesões ateroscleróticas nas paredes dos vasos.^2^

Na maioria dos casos, o *kinking* vascular é assintomático, mas pode haver sintomas neurológicos, como ataque isquêmico transitório (AIT), dependendo do efeito hemodinâmico da estenose arterial na perfusão.^1,4^

A investigação diagnóstica é realizada por meio de dados da anamnese, exame físico, e complementada com doppler de carótidas e vertebrais, angiotomografia computadorizada (ATC), angiografia ou angiorressonância magnética (ARM).^5^ O tratamento pode incluir terapia antiagregante e anticoagulante, anti-hipertensivos, estatinas, terapia de vasodilatação cerebral e tratamento cirúrgico, se indicado.^3^

O *kinking* é mais comum na artéria carótida interna, mas é raro nas artérias subclávia e carótida comum,^4,5^ e não há registros na literatura de casos simultâneos nesses vasos. O objetivo desse trabalho é descrever um caso de *kinking* vascular nas artérias subclávia e carótida comum, diagnosticado em um pronto-socorro de um hospital particular na cidade do Rio de Janeiro.

## Relato de Caso

Paciente do sexo feminino, 77 anos, deu entrada no pronto-socorro por disartria e paresia de membros superiores enquanto tomava café da manhã, com melhora espontânea após 15 minutos. Previamente portadora de HAS, DM, dislipidemia, doença de Parkinson e AIT.

À admissão, os sinais vitais mostraram pressão arterial de 203x106 mmHg, frequência cardíaca de 78 bpm, frequência respiratória de 18 irpm, saturação de oxigênio periférica de 98% e temperatura axilar de 36 °C.

Exame neurológico revela paciente acordada, desorientada, com miose e ptose à direita, sem déficit de força.

**Figura 1:**
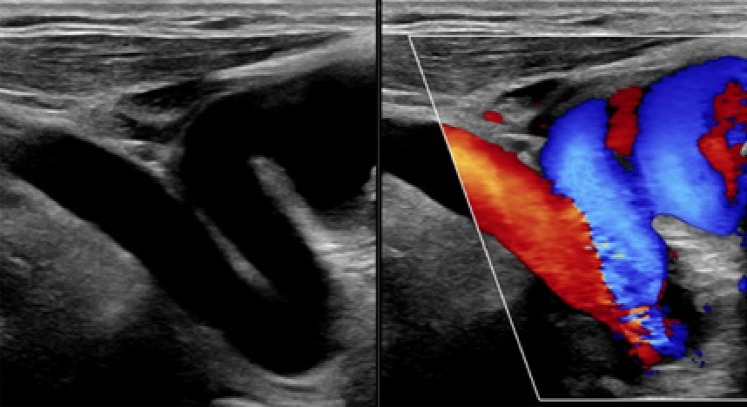
– Doppler arterial de artérias carótidas e vertebrais esquerdas, mostrando um kink em origem de carótida comum.

O doppler arterial da subclávia direita mostrou fluxos preservados, com um *kink* anatômico em sua origem. O ecodoppler das artérias carótidas e vertebrais esquerdas mostrou um *kink* em origem de carótida comum, com um aumento significativo de velocidade de pico sistólica na angulação mais aguda (Figura 1); os demais fluxos mantiveram seus valores e morfologia.

**Figura 2:**
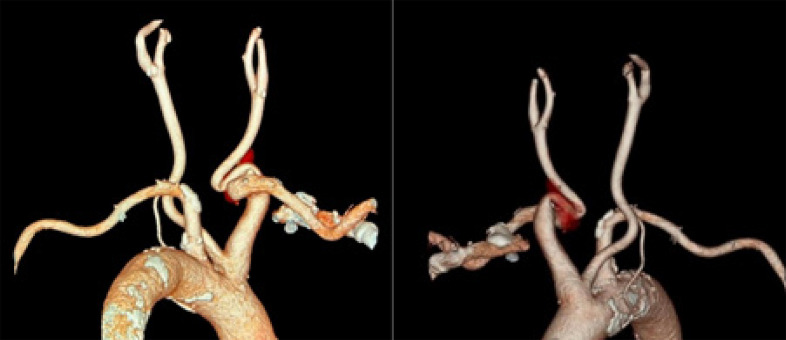
– Angiotomografia computadorizada 3D do pescoço, mostrando kinking na artéria subclávia direita e na artéria comum direita.

**Figura 3:**
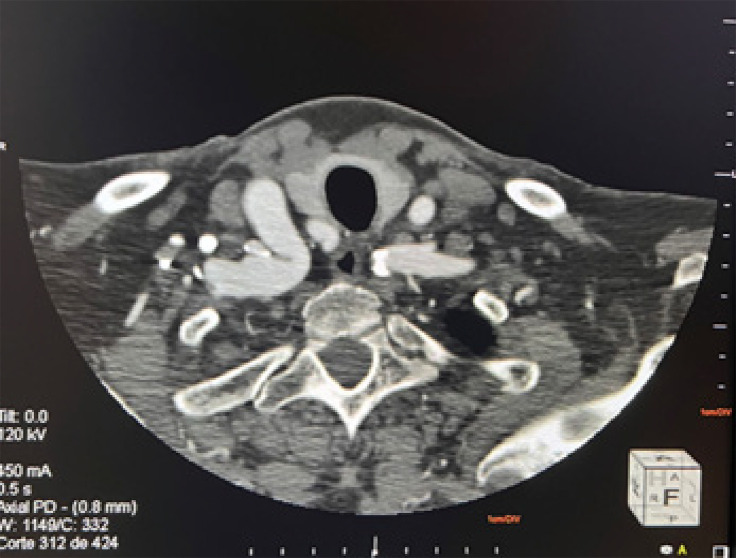
– Tomografia computadorizada de crânio e pescoço, mostrando kinking na artéria subclávia direita.

A tomografia e angiotomografia de crânio e pescoço demonstraram um arco aórtico alongado, tipo II, ateromatoso. Tronco braquiocefálico, artérias carótidas e subclávias foram descritos como ateromatosos, alongados e tortuosos (Figura 2). Confirmou-se também o acotovelamento nas origens das artérias carótida comum e subclávia direitas (Figura 3). Foi observada também uma placa mista na origem da carótida comum esquerda, determinando estenose leve. As carótidas e os ramos apresentaram ateromas difusos bilateralmente, com anatomias preservadas. Foi identificada hipoplasia do segmento A1 da artéria cerebral anterior direita e do segmento P1 das artérias cerebrais posteriores. Outra variação anatômica encontrada foram os demais segmentos das artérias cerebrais posteriores que recebiam irrigação das artérias comunicantes posteriores correspondentes com espessura aumentada. Não foram observadas evidências de malformação vascular ou aneurismas aparentes.

A ressonância e angioressonância magnéticas de crânio indicaram gliose por micorangiopatia (Fazekas 3). Os achados são compatíveis com o histórico e idade da paciente.

O tratamento incluiu ácido acetilsalicílico, clopidogrel, atorvastatina, e controle pressórico e glicêmico. Houve regressão dos sintomas em algumas horas e alta hospitalar em 11 dias. A paciente permanece sob tratamento conservador pela equipe multiprofissional.

## Discussão

As alterações tortuosas vasculares podem ser categorizadas em três graus: no primeiro grau, o ângulo entre os dois segmentos é de 60° e 90°; no segundo grau, a angulação varia de 30° a 60°; e, no terceiro grau, é inferior a 30°, formando um *kinking* que pode provocar sintomas neurológicos associados à etiologia isquêmica.^1^

Na maioria dos casos, o *kinking* vascular, apesar de reduzir o suprimento sanguíneo para o cérebro ao diminuir a pressão arterial, não leva à isquemia cerebral pela compensação via mecanismo autorregulador na perfusão sanguínea cerebral.^1^ No entanto, pode ocorrer estreitamento do vaso, gerando um fluxo sanguíneo turbulento, e subsequente ulceração e embolização da íntima.^6^ Isso resulta em sintomas semelhantes aos causados pela doença aterosclerótica, como o AIT.^6^ Os sintomas do *kinking* são descritos na literatura como potencialmente desencadeados pela posição da cabeça,^3,7^ principalmente na presença de anormalidades anatômicas, ou em indivíduos com doença aterosclerótica,^2^ o que é uma hipótese para o quadro apresentado pela paciente.

O arco aórtico tipo II, onde a artéria carótida comum esquerda emerge do tronco braquiocefálico em vez do arco aórtico, é a variante congênita mais comum da ramificação do arco aórtico,^5^ representando 10,5% das origens da artéria carótida comum.^8^ Além disso, foram observadas outras variações anatômicas, como os demais segmentos arteriais das artérias cerebrais posteriores que recebiam irrigação das artérias comunicantes posteriores correspondentes com espessura aumentada. Assim, essa paciente apresentava várias alterações anatômicas que, junto com os fatores de risco agravados pela idade e o alongamento dos vasos,^2^ podem explicar a ocorrência do *kinking* com sintomas neurológicos associados à etiologia isquêmica.

Embora a angiografia seja descrita como diagnóstico padrão-ouro,^5^ a ATC tem sido cada vez mais usada.^8^ O doppler vascular é um método sensível para detectar doenças extracranianas, permitindo o registro da anatomia e da alteração de fluxo.^6^ Neste caso, a hipótese inicial foi de um novo episódio de AIT devido a fatores ateroscleróticos, mas o doppler vascular revelou um aumento significativo na velocidade de pico sistólica na angulação mais aguda, levando à suspeita de *kinking* vascular. Essa suspeita foi posteriormente confirmada pela ATC.

O tratamento pode envolver abordagens cirúrgicas e/ou terapia farmacológica otimizada. A modificação dos fatores de risco sempre é recomendada em pacientes assintomáticos devido à associação com aterosclerose.^9^ No seguimento clínico, é indicada a prescrição de antiagregantes, anticoagulantes (em casos selecionados), anti-hipertensivos, hipolipemiantes e terapia vasodilatadora cerebral. O tratamento invasivo é reservado para pacientes com sintomas de isquemia cerebrovascular ou sinais neurológicos. A angioplastia é o tratamento inicial de preferência,^8^ mas em casos em que não é adequada ou bem-sucedida, a endarterectomia pode ser considerada, embora apresente maior risco em comparação com uma abordagem endovascular.^7,10^ No entanto, no caso relatado, após avaliação pela equipe multidisciplinar, optou se inicialmente pelo tratamento conservador, levando em consideração a idade da paciente, suas comorbidades e a anatomia desfavorável.

Este caso destaca a importância de uma investigação completa, que inclui a anamnese, exame físico detalhado e o uso da multimodalidade de imagem para o diagnóstico e tratamento. Também destaca a documentação de algumas variações anatômicas observadas na mesma paciente.

## Conclusão

O *kinking* vascular representa um desafio diagnóstico e terapêutico na rotina médica. Os sintomas dessa anormalidade são mais evidentes quando há comprometimento da autorregulação da hemodinâmica cerebral, o que pode acontecer no grau 3 ou quando mais de uma artéria é afetada, podendo resultar em sintomas neurológicos associados à etiologia isquêmica.

O diagnóstico requer uma investigação completa, combinando informações clínicas e de várias modalidades de imagem, além da análise de possíveis fatores de risco e predisposição genética, fundamentais para uma melhor compreensão da doença e para realizar tratamento ideal.
