# Supplementary material for: Ataque Isquêmico Transitório em Paciente com Kinking Vascular de Subclávia e de Carótida Comum Direitas - Um Relato de Caso
Source: Arq Bras Cardiol. 2024 Jul 31;121(7):e20230520. [Article in Portuguese] doi: 10.36660/abc.20230520 (PMC11341209; doi:10.36660/abc.20230520)
Supplement: Translation

# Translation: Transient Ischemic Attack in a Patient with Vascular Kinking of the Subclavian and Right Common Carotid Arteries - A Case Report

**DOI:** 10.36660/abc.20230520i

**Authors:** Katia Gleicielly Frigotto, Washington Luiz Batista da Costa, Lais Antonucci Ferreira, Daniela Roberta Alves Silva, Giovana Salviano Braga Garcia, Luciano de Figueiredo Aguiar, Bruno de Souza Paolino

**Affiliations:** 1 Brazil Hospital São Lucas Copacabana, Rio de Janeiro, RJ – Brazil; 2 Brazil Universidade do Grande Rio Professor José de Souza Herdy, Rio de Janeiro, RJ – Brazil

**Keywords:** Vascular Diseases, Cerebrovascular Disorders, Transient Ischemic Attack

## Introduction

Vascular kinking is a vascular angular change where an acute angle is formed in an artery.^1^ Both embryogenic and acquired causes can lead to this change. Studies indicate that atherosclerosis, hypertension, and aging are significant contributors to carotid abnormalities.^2^ Other factors such as trauma, congenital causes, or inflammatory diseases are uncommon and typically associated with atherosclerosis.^3^ Congenital factors may manifest clinically later in life, being exacerbated by conditions like systemic arterial hypertension (SAH), diabetes mellitus (DM), dyslipidemia, smoking, rheumatological disorders, and heart diseases.^2^

There are two types of kinking. The first one being congenital, most prevalent in women in their late 40s and early 50s, being clinically significant in advanced age. The second type is linked to atherosclerotic risk factors, such as SAH, dyslipidemia, DM, and smoking.^1^ However, determining the exact role of carotid abnormalities in causing ischemic symptoms is challenging due to their frequent association with atherosclerotic lesions in vessel walls.^2^

In most cases, vascular kinking is asymptomatic, but neurological symptoms, such as transient ischemic attack (TIA), may arise depending on the hemodynamic effect of arterial stenosis on perfusion.^1,4^

The diagnostic investigation includes gathering data from patient history and physical examination, supplemented by carotid and vertebral Doppler ultrasound, computed tomography angiography (CTA), angiography, or magnetic resonance angiography (MRA).^5^ Treatment may include antiplatelet and anticoagulant therapy, antihypertensives, statins, cerebral vasodilation therapy, and surgical treatment, as indicated.^3^

Although kinking predominantly affects the internal carotid artery, occurrences in the subclavian and common carotid arteries are rare.^4,5^ There are no reports in the literature of simultaneous cases in these vessels. This study aims to describe a case of vascular kinking involving the subclavian and common carotid arteries, diagnosed in the emergency room of a private hospital in Rio de Janeiro.

## Case Report

A 77-year-old female patient was admitted to the emergency room reporting dysarthria and paresis of the upper limbs while eating breakfast, with spontaneous improvement after 15 minutes. Patient previously had SAH, DM, dyslipidemia, Parkinson’s disease and TIA.

On admission, vital signs showed a blood pressure of 203x106 mmHg, heart rate of 78 bpm, respiratory rate of 18 breaths per minute, peripheral oxygen saturation of 98%, and axillary temperature of 36 °C.

Neurological examination revealed the patient to be awake, disoriented, with miosis and ptosis on the right side, and no strength deficit.

The right subclavian arterial Doppler showed preserved flows, with anatomical kink in its origin. Doppler ultrasound of the left carotid and vertebral arteries showed common carotid origin kinking, with significantly increased systolic peak velocity at the most acute angulation (Figure 1); other flows preserved their values and morphology.

**Figure 1:** – Arterial Doppler of left carotid and vertebral arteries showing common carotid origin kinking.

CT and angiotomography of the skull and neck revealed elongated, type II, atheromatous aortic arch. Brachiocephalic trunk, carotid and subclavian arteries were atheromatous, elongated and tortuous (Figure 2). Images also confirmed the kinking at the right common carotid and subclavian arteries origins (Figure 3). A mixed plaque was also described at the origin of the left common carotid artery, determining mild stenosis. Carotids and branches showed diffuse atheroma bilaterally, with preserved anatomies. Hypoplasia of the A1 segment of the right anterior cerebral artery and the P1 segment of the posterior cerebral arteries was identified. Another anatomical variation observed was the posterior cerebral arteries receiving increased blood supply from corresponding posterior communicating arteries with enhanced thickness. There was no evidence of vascular malformation or apparent aneurysms.

**Figure 2:** – 3D computed tomography angiography of the neck showing right subclavian artery and right common artery kinking.

**Figure 3:** – Computed tomography of the skull and neck showing right subclavian artery kinking.

Cranial magnetic resonance and angiography indicated gliosis due to mycorangiopathy (Fazekas 3). Findings are compatible with the patient’s history and age.

Treatment included acetylsalicylic acid, clopidogrel, atorvastatin, and management of blood pressure and glycemic levels. Symptoms regressed within a few hours, and the patient was discharged after in 11 days. She continues under conservative treatment by the multidisciplinary team.

## Discussion

Vascular crooking can be categorized into three degrees: in the first degree, the angle between the two segments is 60° and 90°; in the second degree, the angulation ranges from 30° to 60°; and in the third degree, it is less than 30°, forming a kinking that may cause neurological symptoms associated with ischemic etiology.^1^

In most cases, vascular kinking, despite reducing blood supply to the brain through lowering blood pressure, does not lead to cerebral ischemia through compensation via an autoregulatory mechanism in cerebral blood perfusion.^1^ However, narrowing of the vessel may occur, thereby creating turbulent blood flow, and subsequent ulceration and embolization of the intima.^6^ This result in symptoms similar to those caused by atherosclerotic disease, such as TIA.^6^ Kinking symptoms are described in the literature as potentially triggered by the position of the head,^3,7^ especially in the presence of anatomical abnormalities, or for individuals with atherosclerotic disease,^2^ which is a hypothesis for the patient’s condition.

The type II aortic arch, where the left common carotid artery originates from the brachiocephalic trunk rather than directly from the aortic arch, is the most common congenital variant of aortic arch branches,^5^ accounting for 10.5% of cases.^8^ Moreover, other anatomical variations were observed, such as the posterior cerebral arteries receiving increased blood supply from corresponding posterior communicating arteries with enhanced thickness. Thus, this patient presented several anatomical changes, which may justify, together with the risk factors aggravated by age and associated with the elongation of the vessels,^2^ the occurrence of kinking with neurological symptoms associated with ischemic etiology.

Although angiography is described as the diagnostic gold standard,^5^ CTA has been increasingly used.^8^ Vascular Doppler imaging proves to be a sensitive method for detecting extracranial vascular diseases, allowing for the monitoring of anatomical and flow alterations.^6^ In this case, the initial hypothesis was a new episode of TIA due to atherosclerotic factors, but vascular Doppler revealed a significant increase in systolic peak velocity at the most acute angulation, raising suspicion of vascular kinking. This suspicion was later confirmed through CTA.

Treatment may involve surgical approaches and/or optimized pharmacological therapy. Modification of risk factors is always recommended to asymptomatic patients, due to the association with atherosclerosis.^9^ In clinical follow-up, treatment typically includes antiplatelet agents, anticoagulants (in selected cases), antihypertensive medications, lipid-lowering drugs, and cerebral vasodilators. Invasive treatments are generally reserved for patients with cerebrovascular ischemia symptoms or neurological signs. Angioplasty is the first-line treatment,^8^ but in cases where it is not adequate or successful, endarterectomy may be considered, although the risk is greater when compared to an endovascular approach.^7,10^ However, in the case reported herein, after evaluation by the multidisciplinary team, conservative treatment was initially chosen, due to the patient’s age, comorbidities, and unfavorable anatomy.

This case underscores the critical role of comprehensive investigation, including patient history, detailed physical examination, and multimodal imaging, in both diagnosing and treating the condition, as well as emphasizing the documentation of anatomical variations observed within the same patient.

## Conclusion

Vascular kinking presents a significant diagnostic and therapeutic challenge in routine medical practice. Symptoms of this condition are particularly pronounced when autoregulation of cerebral hemodynamics is compromised, often occurring in grade 3 cases or when multiple arteries are affected, potentially leading to neurological symptoms of ischemic origin.

Comprehensive diagnosis involves thorough investigation, integrating clinical data with various imaging techniques, and analyzing potential risk factors and genetic predispositions, which are essential steps for a deeper understanding of the disease and for delivering optimal treatment strategies.
